# Review of Simulation in Pediatrics: The Evolution of a Revolution

**DOI:** 10.3389/fped.2015.00106

**Published:** 2015-11-30

**Authors:** Rahul Ojha, Anthony Liu, Deepak Rai, Ralph Nanan

**Affiliations:** ^1^Schulich School of Medicine and Dentistry, Western University, London, ON, Canada; ^2^Sydney Medical School Nepean, The University of Sydney, Sydney, NSW, Australia

**Keywords:** simulation, pediatric simulation, graduate medical education, undergraduate medical education, mannequins

## Abstract

Recent changes in medical education have highlighted the importance of experiential learning. Simulation is one model that has gained significant attention in the last decade and has been widely adopted as a training and assessment tool in medical education. Pediatric simulation has been utilized to teach various skills including resuscitation and trauma management, procedural skills, and team training. It is also a valuable tool for health care educators, as it allows learners to achieve competence without putting patients at risk. Recent literature demonstrates increased retention of knowledge and skills after simulation-based training. Further research is required to improve current simulation curriculums, develop validated assessment tools, and to demonstrate improved clinical outcomes after simulation-based training. We conducted an online search of original and review articles related to simulation and pediatric medical education and provide an overview of the role and utility of simulation in pediatrics.

**Key Points**
- Simulation in pediatrics has been widely accepted and adapted as a training and assessment tool in medical education.
- Simulation in pediatrics has been utilized to teach various skills including resuscitation and trauma management, procedural skills, and team training.
- Further research is required to improve current simulation curriculums, to develop validated assessment tools, and to demonstrate improved clinical outcomes after simulation-based training.

## Introduction

This review explores evidence of the role and utility of simulation in pediatrics.

It highlights the role of simulation as an educational tool with particular emphasis on resuscitation, procedural skills, crisis resource management (CRM), and team training. It also focuses on avenues for future research in the field of pediatric simulation. This review will focus on the use of patient simulators and task trainers.

Medical education has traditionally relied on an apprenticeship model to educate learners. In this model, the physician learners observe experienced physicians perform a skill on a patient that they then perform themselves (1). Studies have shown deficiencies in knowledge and retention of skills among medical trainees who are trained via this method (2, 3). Changing patterns in the delivery of health care (shorter hospital stays for patients, more focus on preventative care) and an increasing number of trainees in medical school and postgraduate programs have led to decreased trainee exposure to less common illnesses as well as acute medical situations. Changes in trainee work hours, specifically limitations to the duration of call shifts, have decreased resident clinical exposure (4).

New approaches to medical education are required to enhance learning opportunities. Simulation has been used as a teaching tool for many decades in various fields, including military training and the aviation industry (5). David Gaba, one of the pioneers of modern day medical simulation, defined simulation as “an instructional process that substitutes real patient encounters with artificial models, live actors, or virtual reality patients” (6). Simulation can take many different forms, including role playing, use of standardized patients, part-task-trainers (either physical or virtual reality), computer patients (a screen based “virtual world”), or electronic patients. Standardized patients allow trainees to practice communication and interpersonal skills. Part-task-trainers and virtual reality simulators are best practiced in a dedicated skills laboratory and allow for skill repetition and practice. The electronic patient simulators are computer driven and fully interactive mannequins with varying levels of fidelity. Low fidelity mannequins offer a physical body, but require an instructor to provide physiological information. High-fidelity mannequins are interactive and life-like and can illustrate physiological conditions. Mannequin-based simulation can also be used in a skills laboratory, although the more complex recreations of clinical tasks require fully equipped simulation centers or the ability to bring the simulator into an actual work setting (*in situ* simulation) (6, 7).

There has been a significant growth and acceptance of medical simulation over the last two decades. There is increasing evidence that simulation training improves health care education, practice, and patient safety (8). It is a tremendous tool for health care educators, as it allows learners to achieve competence without putting patients at risk. The literature suggests that simulation in medical education improves the knowledge base, skills, and teamwork of students and clinicians, both in resuscitation and procedural skills (9). Pediatric educators have utilized simulation in all of these capacities.

## Methods

A literature search was performed to identify articles relevant to the role of simulation in pediatrics. A search of Medline, CINHAL, Cochrane Database, and PubMed was conducted to identify articles relevant to simulation, and its use in pediatrics. Inclusion criteria included the following:

(1) Utilization of simulation in pediatrics as an educational intervention with measured outcomes;
(2) Articles related to human patient simulators or task-trainers;
(3) Accessibility of full text articles published in English language in peer-reviewed journals.

**Figure 1:**
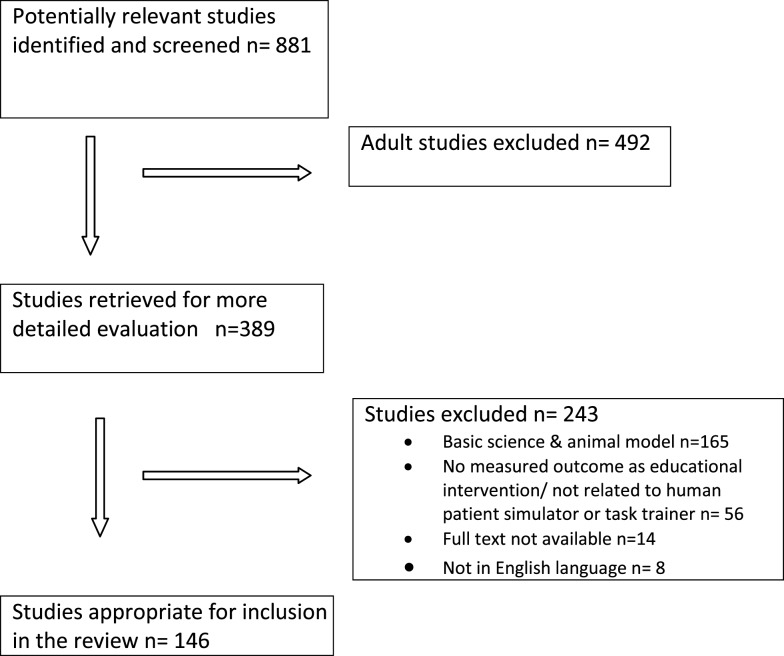
**Selection process for study inclusion**.

References from relevant articles were then explored, and appropriate citations were also reviewed and included. Each abstract was initially read by two reviewers to identify whether the article was likely to fulfill the inclusion criteria. The titles that had no relevance to the review (basic science and animal models) were excluded from the review. A total of 146 articles were included in this review. The process for selection of articles for review is shown in a flow diagram (Figure 1).

## Simulation in Pediatrics

In the last decade, there has been significant focus on realistic pediatric simulators (specific task-trainers and patient mannequins) and increasing numbers of studies evaluating the effectiveness of simulation in pediatrics as an educational tool. Many pediatric residency programs across the globe have started using simulation as a method for procedural and resuscitation education. It has been used in various aspects of pediatric training, including resuscitation and trauma management, procedural skills, and CRM and team training (10).

## Resuscitation and Trauma Training

Historically, pediatric residents, during their training, have acquired most of their experience in resuscitation and procedural skills through direct patient contact in the emergency department, intensive care unit, and inpatient wards (11). It is reported that pediatric cardiopulmonary resuscitations are more infrequent than similar events in adults with potentially good outcomes if patients are successfully resuscitated (12). Resuscitation training programs, such as Advance Pediatric Life Support (APLS), Pediatric Advanced Life Support (PALS), Neonatal Resuscitation Program (NRP), and other equivalent resuscitation program, have become a bench mark for CPR training and are mandatory training programs for pediatric acute care providers, and require re-certification every 2–3 years (13). Regardless of current educational practices, studies have shown that majority of pediatric residents complete their training with insufficient knowledge and experience in the care of critically ill children (2, 14). A survey done by Hunt et al. reported that a large number of postgraduate year 3 (PGY 3) residents had never led a resuscitation event (15). Furthermore, resuscitation courses are insufficient in ensuring that pediatric residents have prolonged mastery of resuscitation skills, as retained knowledge, skills, and confidence have been shown to decline within a few weeks to several months after completion of a resuscitation program (16, 17).

Multiple studies have shown that simulation can improve pediatric resident performance during resuscitations. In a recent study, McKittrick et al. looked at the role of simulated training in resuscitation by providing two cycles of real time simulated training episodes to health care professionals in the pediatric critical care unit. They reported enhanced preparedness for participation in pediatric resuscitation after the training (18). Following a somewhat similar footstep, with the common goal of enhancing residents’ skills at handling emergent pediatric conditions, the concept of a “mock code” as put forward by Andreatta et al. was instituted as an element of residency training. Prior to any indication, these drills were conducted at a few random time periods within a month which formed a subsection of a longer, 4-year cycle. A substantial 17% increase in the cardiopulmonary arrest survival rates was observed within a space of 12 months, which was simply a smaller cross-section of the general positive trend observed in the hospital over 4 years (19).

In addition to resuscitation, pediatric trauma has been identified as an uncommon event that requires practice in managing (20). A multicenter study conducted in 35 pediatric emergency departments in the US identified deficiencies in the stabilization of children presenting to the emergency department with trauma. They found errors throughout all sites and concluded that there is potential to improve trauma stabilization performance using simulation technology (17). Hunt et al. conducted a pediatric trauma simulation in 18 emergency departments across North Carolina. A 6-month follow-up trauma scenario was performed to look at the retention of skills and knowledge. This study demonstrated markedly improved performance by pediatric emergency care providers (21). Other studies have organized trauma simulation training for multidisciplinary pediatric trauma teams and found significant improvement in overall performance after the simulation training (22, 23). There is substantial evidence to suggest that simulation improves performance and team management in resuscitation and trauma situations.

## Procedural Skills

Procedural skills training is an important component of general pediatrics training (24). The Accreditation Council for Graduate Medical Education (ACGME) in the US recommends that pediatric residents gain broad procedural competence during pediatrics training (25). Proficient and appropriate use of procedural skills is an accreditation requirement for pediatric training programs in Canada and Australia (26, 27). In 2007, the Residency Review Committee (RRC) for Pediatrics in US published a list of procedures and skills in which all residents must have “sufficient” training (25). Based on these requirements and additional recommended procedures, Gaies et al. surveyed the opinions of pediatric residency program directors regarding the importance of these procedures and residents’ perceived competency. Notably, a large percentage of program directors did not assign the same level of importance as the RRC did for its’ recommended procedures. They also found that numerous residents failed to achieve procedural skills competence at the end of their training, and the program directors’ assessments of their own residents’ competencies were lower than the RRC goal (3).

The lack of competency in these skills can be justified by several potential reasons. The chief among these reasons are the enhanced skillset of non-physician clinicians and the advancement of ancillary services such as intravenous access teams. These teams/personnel take away from the procedures that were habitually conducted by residents. This was strengthened by Gaies et al. who reported that 12 and 13% of respondents identified residents as the people who perform the majority of venipunctures and intravenous catheter placements, respectively, in the hospitals they rotate (3). The limited opportunities presented to trainees to grasp and apply a procedure to build competence does not astonish due to the time consuming nature of paperwork and other documentation.

As a result, the outlook of competency for emergent and intricate procedures has been reevaluated, with the emphasis placed on the importance of frequent practice to counter intermittently performed procedures (28). Attaining complex procedural skills requires the balanced involvement of several facets including psychomotor, clinical judgment, communication, decision-making, and patient-focused interaction abilities and cannot be brought to fruition through clinical exposure alone (29). In addition, training should be backed by direct observation, frequent practice, and feedback (30). Simulation, therefore, is an ideal method to learn these skills (31).

Studies using simulation task-trainers to teach pediatric residents procedures such as central venous catheter (CVC) insertion, chest tube insertion, and endotracheal intubation are well established in the literature (32–34). Overly et al. evaluated pediatric residents’ abilities to manage an acute airway using high-fidelity medical simulation and identified many areas of concern. They concluded that high-fidelity medical simulation could not only assess a resident’s ability to manage an airway but also be an effective tool in teaching the skills necessary to manage an airway (35). In another study, Thomas et al. described their efforts to train pediatric residents in CVC placement and demonstrated improved performance by pediatric residents immediately following didactic and hands-on training on simulators. Trainee performance, however, substantially decayed by 3-month follow-up (36).

## Crisis Resource Management and Team Training

Resuscitation events in children are uncommon, yet appropriately managing resuscitation is a core competency required by all pediatric trainees (37). The reality of medical crises being less common in pediatrics places residents at risk of being unprepared, hesitant, and highly anxious when such events do arise (38). Sound technical skills are mainstay for managing critically ill patients; however, recent focus has been on combination of technical skills along with cognitive and complementary non-technical skills (39). It is becoming increasingly recognized that principles of crises resource management (CRM) have a role in non-technical skills, including leadership, team building, problem solving, situational awareness, communication skills, and resource management (40). A review by Cheng et al. highlighted the role of CRM for efficient team functioning and subsequent error reduction in high-stakes environments such as acute care pediatrics. They emphasize the role of simulation in crises resource management and highlight the potential for tremendous improvements in patient safety and outcomes, although this has yet to be clearly demonstrated (41).

There are little data available in literature looking at resuscitation team leadership skills in pediatrics. The non-technical and leadership skills, although important in the management of acutely ill children, are not acquired passively during residency, and hence, may require specific education (41). Gilfoyle et al. developed a simulation-based intervention on the principles of CRM and evaluated immediate and long-term learning outcomes to determine whether pediatric residents could acquire and retain team leadership skills in pediatric advanced resuscitation. The study showed that residents were able to acquire resuscitation team leadership skills following the intervention (42). Effect of teaching CRM as a function of residents’ performance in a simulated scenario was investigated in a prospective study by Blackwood et al. who examined the converging and contrasting outcomes of individual residents who were exposed to 1 h of CRM instructions. The findings indicated an improvement in time to “critical initial steps of pediatric resuscitation” and likewise, proficiency in residents’ performance in a simulated scenario (43).

## Future Research

Recent changes in medical education have highlighted the importance of simulation as a form of experiential learning. The utility of simulation in the field of medicine has been suggested for conditions that are rare and critical in nature and require the maintenance of specialized skill and preparedness. However, the interval between initial training and actual use of skills is critical for maintaining the knowledge and skills essential for improving patient outcomes.

There is paucity of literature addressing such strategies that could help in retaining knowledge, skills, and confidence in managing pediatric emergencies. One intervention postulated to be helpful is the spacing effect. This refers to educational encounters, which are spaced and repeated over time (spaced distribution), resulting in more efficient and improved retention of learning, compared with more concentrated educational encounters (44, 45). Ojha et al. designed a pediatric critical care scenario management program using spaced education model to look at the impact of repeated observation of routinely scheduled demonstrations of critical pediatric illness scenarios. These demonstrations were observed by pediatric medical and nursing staff every other week for 3 months, and then the same scenarios were repeated over the next 3-month period. The study found that the implementation of repeated, time-spaced demonstrations provided an effective method for improving knowledge and increasing confidence among pediatric health care professional managing critically ill children (46). Further research is needed to consolidate this approach, determine the appropriate amount of time between sessions and assess the long-term effects of this intervention.

More research is needed on the use of simulation for assessment. Many hospitals and medical schools have begun using high-­fidelity simulation medicine in their educational curriculum. Several studies have used simulation-based assessment tools and have found that the simulation-based assessments are valid and reliable measures of clinical performance and resident competency (47, 48). The reliability and validity of a scoring instrument aimed to evaluate clinical performance during simulated resuscitations were examined by Donoghue et al. The parameters used to measure performance in this study, in which the residents engaged, were based on timing, sequence, and quality and were judged and scored individually. The instrument’s ability to measure clinical performance during simulated scenarios was reinforced through the reliable and valid scores (49). In an attempt to ascertain whether resident scores could be utilized to authenticate the reliability and validity of a multiple-scenario assessment, McBride et al. developed a bank of 20 simulated pediatric crisis scenarios. Every resident was engaged in 10 scenarios that were scored using an action-item checklist and a global score by 2 individual assessors. The results revealed that the resident scores were legitimate tools for the measure of performance using simulation (50).

The clinical significance of measured performance in a simulated setting, however, remains uncertain. Additional evidence is needed, including studies to determine if this type of training improves physicians’ management of real-life critical events. This ties into a third area requiring future research: demonstrating an improvement in patient safety and/or outcomes with simulation training. The study by Andreatta et al., which showed an increase in cardiopulmonary arrest survival rates after the institution of a simulated mock code program, is a rarity (19). A study by Knight et al. did show an increase in survival to hospital discharge after the introduction of simulation-based composite team training (51). While there is rapidly growing interest and enthusiasm for simulation as a method of optimizing training and patient safety, evidence clearly documenting a positive effect on patient safety and improved patient outcomes remains elusive (52), and it is an area where significantly more research is required.

## Conclusion

Simulation has tremendous potential as a teaching and assessment tool for pediatric acute care providers. Advances in simulation education continue to emerge for pediatric medicine. Simulation has been used to teach resuscitation and trauma management skills, procedural skills, and CRM to pediatric trainees. Future research must explore ways to maximize the impact of training sessions to improve retention, the use of simulation for assessment, and finally to assess the impact of simulation training on outcomes in the pediatric population.

## Conflict of Interest Statement

The authors declare that the research was conducted in the absence of any commercial or financial relationships that could be construed as a potential conflict of interest.
